# Metabolite Biomarkers of Prolonged and Intensified Pain and Distress in Head and Neck Cancer Patients Undergoing Radio- or Chemoradiotherapy by Means of NMR-Based Metabolomics—A Preliminary Study

**DOI:** 10.3390/metabo14010060

**Published:** 2024-01-17

**Authors:** Łukasz Boguszewicz, Alicja Heyda, Mateusz Ciszek, Agata Bieleń, Agnieszka Skorupa, Jolanta Mrochem-Kwarciak, Krzysztof Składowski, Maria Sokół

**Affiliations:** 1 Department of Medical Physics, Maria Sklodowska-Curie National Research Institute of Oncology, Gliwice Branch, 44-102 Gliwice, Poland; mateusz.ciszek@gliwice.nio.gov.pl (M.C.); agnieszka.skorupa@gliwice.nio.gov.pl (A.S.); maria.sokol@gliwice.nio.gov.pl (M.S.); 2 1st Radiation and Clinical Oncology Department, Maria Sklodowska-Curie National Research Institute of Oncology, Gliwice Branch, 44-102 Gliwice, Poland; alicja.heyda@gliwice.nio.gov.pl (A.H.); agata.bielen@gliwice.nio.gov.pl (A.B.);; 3 Analytics and Clinical Biochemistry Department, Maria Sklodowska-Curie National Research Institute of Oncology, Gliwice Branch, 44-102 Gliwice, Poland; jolanta.mrochem-kwarciak@gliwice.nio.gov.pl

**Keywords:** HNSCC, radiotherapy, chemotherapy, distress, pain, metabolomics

## Abstract

Treatment of head and neck squamous cell carcinoma (HNSCC) has a detrimental impact on patient quality of life. The rate of recognized distress/depression among HNSCC patients ranges from 9.8% to 83.8%, and the estimated prevalence of depression among patients receiving radiotherapy is 63%. Shorter overall survival also occurs in preexisting depression or depressive conditions. The present study analyzes the nuclear magnetic resonance (NMR) blood serum metabolic profiles during radio-/chemoradiotherapy and correlates the detected alterations with pain and/or distress accumulated with the disease and its treatment. NMR spectra were acquired on a Bruker 400 MHz spectrometer and analyzed using multivariate methods. The results indicate that distress and/or pain primarily affect the serum lipids and metabolites of energy (glutamine, glucose, lactate, acetate) and one-carbon (glycine, choline, betaine, methanol, threonine, serine, histidine, formate) metabolism. Sparse disturbances in the branched-chain amino acids (BCAA) and in the metabolites involved in protein metabolism (lysine, tyrosine, phenylalanine) are also observed. Depending on the treatment modality—radiotherapy or concurrent chemoradiotherapy—there are some differences in the altered metabolites.

## 1. Introduction

Head and neck squamous cell carcinoma (HNSCC) and its treatment have a detrimental impact on many aspects of health-related quality of life. Most studies on the quality of life of HNSCC survivors have shown that patients suffer from distress, depression, pain and a wide variety of persistent cancer-related symptoms. The long list of patient issues includes poor physical and psychosocial functioning, persisting pain in the head, neck and shoulder area, fatigue, insomnia, problems with chewing, swallowing, speaking, mouth dryness, taste alteration, malnutrition, mood, body image concerns, intimate relationships, and financial issues [1,2,3,4]. The rate of recognized distress/depression among HNSCC patients may range from 9.8% to 83.8%, and the pooled estimated prevalence of depression among patients receiving radiotherapy is 63% with a significant heterogeneity among the studies [5]. HNSCC patients are twice as likely to commit suicide compared to other cancer patients [6] or seven times more compared to the general population [7,8]. The areas of patient experience, such as poor overall quality of life, pain, eating and speech problems, are associated with survival in head and neck cancer (HNC) [9,10,11,12]. Shorter overall survival also occurs in pre-existing depression or depressive conditions [13,14].

The experience of persisting pain dominates among other symptoms despite using painkillers and coming to follow-up visits. A meta-analysis of 82 studies evaluating pain in HNC patients treated according to various protocols estimated the incidence of any pain in 57% of patients before treatment and 42% after the treatment [15].

Musculoskeletal pain and psychological distress have frequently been shown to be associated with fatigue across clinical conditions and in the general population and may represent transdiagnostic mechanisms for maintenance and exacerbation of fatigue [16]. Chronic stress is an overwhelming, exhausting experience that leads to dysregulation of the hypothalamic–pituitary–adrenal (HPA) axis. It causes an increase in cortisol, norepinephrine and epinephrine, which are catecholamines released from the adrenal medulla and the neurons of the sympathetic nervous system [17,18]. Distress affects numerous pathways of tumorigenesis, tumor proliferation and metastasis [19]. Many patients with chronic stress and depression have higher levels of multiple inflammatory markers, including the cytokine Interleukin 6 (IL-6) [20,21,22,23]. IL-6 concentration and expression are the notable factors specifically in oral squamous cell carcinoma (OSCC). One in vitro study has shown that norepinephrine at a concentration compatible with physiological stress levels in humans can upregulate IL-6 expression and induce OSCC cell proliferation [21]. Chronic stress experience in a mouse model also promoted oral cancer growth and angiogenesis with increased circulating catecholamine and glucocorticoid levels [22]. A meta-analysis of 99 studies confirmed that IL-6 plays several key roles in different aspects of HNSCC, such as carcinogenesis, progression, invasiveness, angiogenesis and metastasis [23].

Thus, distress and pain management and measurement during anticancer therapy is an area of great clinical importance, and identifying stress and pain-related metabolic factors is beneficial for the optimisation of patient treatment and wellbeing [24]. The measures of pain intensity and pain distress, like the Numeric Pain Rating Scale (NPRS) and the stress-rating scale from the Distress Thermometer (DT), capture, however, only part of the pain experience. Age, gender and pain may influence the NPRS and DT scores [25,26,27].

Numerous studies report prevalent metabolic derangements during chronic stress, including energy metabolism (gluconeogenesis, glycogenolysis, thermogenesis in brown adipose tissue), lipid metabolism, fatty acid uptake, glyco-metabolism and amino acid formation and alterations in hormone concentrations [28,29,30,31,32], while chronic pain is associated with inflammation, triglyceride metabolism and increased lactate production [33,34,35,36]. It is known that the metabolic disturbances find their expression systemically, in variations in the levels of the blood plasma and serum metabolites; hence, metabolomics are frequently used for the study of various types of diseases, including chronic and acute pain and distress conditions [37,38,39]. Among the the nuclear magnetic resonance (NMR) -detectable blood serum metabolites, those potentially distress-related include glutamic acid, kynurenine, tryptophan, and 4-hydroxyproline [40,41,42], while the chronic pain conditions, although not related to cancer and its treatment, result in altered concentrations of 2-hydroxybutyrate, 2-hydroxyisocaproate, citrate, isocitrate, choline, glycine, glutamine, isoleucine, phenylalanine, pyroglutamate, apolipoprotein A1 and the free cholesterol to HDL ratio [39,43]. However, the above results often come from single studies or are not confirmed by other studies; therefore, finding reliable and unambiguous biomarkers is still a matter for the future. While the objective biomarkers seem to be more reliable indicators of patient distress, the patient-reported measures of psychosocial distress are also important for clinicians to understand and respond to cancer patient experiences [44].

To the best of our knowledge, this is the first attempt to characterize the pain and distress accumulated with the disease and its treatment in the patients with HNSCC. In these patients, we have previously successfully used NMR-based metabolomics to identify the radiotherapy- [45] and chemotherapy [46]-induced changes in blood serum as well as to describe the metabolic profile of treatment-induced toxicity [47,48]. The objective of the present study is to evaluate whether and to what extent the magnitude and duration of the distress and pain during the anticancer treatment affect the metabolic profile of the blood serum. The obtained results may be helpful in estimating risk factors for HNSCC patients and may contribute to improving their quality of life, being the next step towards a personalized medicine.

## 2. Materials and Methods

### 2.1. Characteristics of the Patient Groups

The retrospective study was approved by the Ethics Committee, and the informed written consent of the participants was obtained. The studied group consisted of 69 HNSCC patients, 52 men and 17 women, all Caucasians, aged between 46 and 74 years (median age 59 years) and treated in the First Radiation and Clinical Oncology Department of the Maria Sklodowska-Curie National Research Institute of Oncology, Gliwice Branch, Poland. The patients’ demographic data are presented in Table 1. All patients were treated with radical intent with radiotherapy (RT) or concurrent radio–chemotherapy (CHRT). Three fractionation techniques were used in RT: CONV (conventional fractionation), CAIR (continuous accelerated irradiation) and SIB (accelerated irradiation with simultaneous integrated boost); CHRT was realized with CONV fractionation. The detailed description of the fractionation schemes and the analysis of their impact on the metabolic profile of blood serum are available in [45]. The RT and CHRT subgroups show significant differences in tumor localization and disease advancement (Table 1).

The patients, qualified for a radical HNSCC treatment, were in good or very good shape, with ZUBROD 0 or 1. The comorbidities in this group had little impact on the expected tolerance of oncological treatment.

During the treatment, the patients were administered non-steroidal anti-inflammatory drugs (NSAIDs) to provide analgesia for patients with moderate pain and opiates/opioids in the case of severe pain. Acute radiation reactions, regardless of their severity, were effectively treated on a day-to-day basis. The symptoms of acute mucositis were alleviated by a supportive treatment and relieved on a somatic level. The intensity of mucositis was controlled; none of the patients had any breaks during the RT or CHRT.

### 2.2. Distress and Pain Measurements

The measurements were carried out immediately before the beginning of RT/CHRT and after each week of treatment, resulting in 8 records for CHRT and 7 for RT, respectively.

The patients were evaluated using the Numeric Pain Rating Scale (NPRS) and the stress-rating scale from the Distress Thermometer (DT) outcome measure every week to obtain the current levels of distress and pain. NPRS is a simple unidimensional measure of pain intensity in adults. It is a numeric segmented vertical bar, a version of the primary Visual Analogue scale of pain, which employs an 11-point scale, ranging from 0 for no pain to 10 for the worst pain imaginable [49]. The NPRS is easy to administer and assess, valid and reliable [50], and appropriate for all adults, as it is not related to the patient’s education [51]. The patients were asked to mark the level of pain in the head and neck area. In turn, the National Comprehensive Cancer Network’s DT is a quick and efficient tool to screen for distress in cancer patients. It consists of a stress rating scale and an additional checklist of 39 symptoms and problems that allows the patients to identify which of the causes, grouped into five categories (practical problems, family problems, emotional problems, spiritual or religious problems, and physical problems) are the root cause of the distress [52,53]. This tool is a numeric segmented vertical bar, rated from 0 to 10 (11 items), ranging from no distress to extreme distress. It is easy to administer and score [52], being sensitive and reliable for distress screening with the recent cut-off scores for clinically significant distress of 3/10 points [54] or 4/10 points [55].

### 2.3. Serum Samples Collection

Overnight fasting blood samples from the peripheral vein were collected at weekly intervals (simultaneous with the distress/pain measurements), from the day before until the last week of the treatment (8 sampling points for CONV RT and 7 sampling points for CAIR/SIB RT). A total of 516 blood samples were collected; however, 12 samples were rejected due to insufficient quantity, giving a total of 504 samples. The samples were incubated for 30 min at room temperature and then centrifuged (1000× *g*, 10 min) to remove the clot and stored frozen at −80 °C until the NMR measurements were performed.

### 2.4. Sample Preparation for NMR Spectroscopy

After two-step thawing (at 4 °C and at room temperature), the serum samples were mixed with a phosphate buffer (pH 7.4) containing D_2_O and Trimethylsilylpropanoic acid (TSP). The aliquots of 600 µL of the solution were transferred into 5 mm Wilmad WG-1235-7 NMR tubes (Wilmad Labglass, Vineland, NJ, USA) and kept at 4 °C until the NMR analysis.

### 2.5. Measurement Protocol and Quality Control

We applied our standard measurement protocol [45,46,47,48]. The ^1^H NMR spectra were acquired on a Bruker 400 MHz Avance III spectrometer (Bruker Biospin, Rheinstetten, Germany) equipped with a 5 mm PABBI probe. The standard quality-control tests (shim quality, resolution, 90° pulse and water suppression) were performed at every measurement day. The NMR probe tuning and matching, shimming, determination of the transmitter offset value for the water pulse presaturation and 90° pulse adjustments were always carried out for each sample. All the experiments were run with the receiver gain set to 90.5 and the temperature to 310 K. For each serum sample, four ^1^H NMR spectra were acquired with the following pulse sequences: Nuclear Overhauser Effect SpectroscopY (NOESY), Carr–Purcell–Meiboom–Gill (CPMG), diffusion-edited (DIFF) and J-resolved (JRES). The characteristics of the spectra as well as the pulse sequence parameters are given in the supplementary material (Table S1).

### 2.6. Spectra Post-Processing

All 1D spectra underwent the following post-processing steps: an exponential line broadening of 3 Hz was applied to all FIDs prior to Fourier transformation (FT), and after FT of the FIDs an automatic phase correction (in Topspin software version 3.1 from Bruker Biospin, Rheinstetten, Germany) was performed; then, the spectra were referenced to the methyl doublet of alanine at 1.5 ppm; the bucketing over the region 9.0–0.5 ppm with the bucket width set to 0.002 ppm (using AMIX software version 4.0.2 from Bruker Biospin) was applied to reduce the number of variables to 3541. The water signal region (5.15–4.38 ppm, d = 0.77 ppm) was excluded. No normalization was applied. This is the standard protocol used in our metabolomic lab [45,46,47,48].

### 2.7. Metabolite Identification

Metabolite identification was performed by a comparison of the CPMG spectra with the reference compounds library (in Chenomx NMR Suite Professional, Chenomx Inc., Edmonton, AB, Canada), as well as by using multiplicity and scalar coupling information extracted from the 2D JRES spectra, information from the Human Metabolome Database (http://www.hmdb.ca/, accessed on 19 November 2023) and the available literature.

### 2.8. Metabolite Quantification

The low-molecular-weight metabolites were quantified from 1D positive projections of the JRES spectra, while the diffusion-edited spectra were used for quantification of the lipid signals. The integrals were estimated in the spectral regions defined individually for each metabolite using “the sum of all points in region” method in AMIX (Bruker Biospin) software version 4.0.2.

### 2.9. Data Analysis and the Validation of the Multivariate Model

The multivariate analyses were carried out using SIMCA (Umetrics, v. 17) software. Orthogonal partial least squares discriminant analysis (OPLS-DA) was used for class discrimination. In this study, two-class models were used, distinguishing the classes defined as H (High) and L (Low). A detailed description of these classes is provided in Section 3.1. The NMR variables were Pareto-scaled. The OPLS-DA models were assessed using permutation testing and ANOVA of the cross-validated residuals (cv-ANOVA). The univariate statistical analyses were carried out using Statistica software (Statsoft, v. 12). In this work, multiple samples from the same patient are analyzed. Therefore, in univariate tests, the medians of relative concentrations of individual metabolites calculated from all measurement points of a given patient are analyzed. All univariate analyzes were performed for 69 independent cases.

## 3. Results

To facilitate the statistical evaluation of the influence of distress and pain on the blood serum metabolic profile, the following four variables were defined:

- WOD (weeks of distress)—the number of weeks during RT/CHRT when the distress was >0;
- MD (maximum of distress)—a maximum value of the distress during RT/CHRT;
- WOP (weeks of pain)—the number of weeks during RT/CHRT when the pain was >0;
- MP (maximum of pain)—a maximum value of the pain during RT/CHRT.

As described in Section 2.2 and Section 2.3, distress and pain were evaluated using a thermometer scale ranging from 0 (no distress/pain) to 10 (the worst distress/pain imaginable). The WOD and WOP variables are ranged between 0 to 8 or 7 (for CHRT and RT, respectively) and denote the number of weeks where distress or pain were >0. The MD and MP variables range between 0 and 10 and denote the max distress or pain value for a particular patient. The median values of these variables were calculated for the whole study group as well as for the RT and CHRT treatment subgroups separately and are listed in Table 2. As our previous studies show, chemotherapy introduces additional (to RT) disturbances in the metabolic profile of blood serum [45]. Therefore, the statistical analyses were performed in the whole study group and in its subgroups.

The medians of the maximum distress and pain values were identical throughout the groups (Table 2), while in the case of a CHRT modality the number of weeks with non-zero distress and pain was higher (without statistical significance). The patients treated with concurrent radiochemotherapy received RT exclusively in a conventional scheme (5 days per week, 7 weeks), whereas RT alone was realized with three different schemes (conventional as well as two accelerated fractionations spread over 6 weeks).

### 3.1. Multivariate Modelling

According to the data presented in Table 2, the patients (and in consequence all samples collected from a particular patient) were divided into two classes:

- L: Low (<median value),
- H: High (≥median value).

If the number of weeks in which a given patient showed stress or pain greater than zero is higher or equal to the median, then all his/her blood serum samples are included in the class H of the analyzed parameter. The same applies to the severity of distress and pain. If the maximum distress or pain for a given patient (at any measurement point) is higher or equal to the median, all his/her blood serum samples are included in class H of the analyzed parameter. In both cases, the remaining samples belong to class L.

The multivariate two-class discrimination models (OPLS-DA) were used in order to identify a potential influence of the distress and pain magnitudes and their duration on the blood serum metabolic profile.

**Figure 1:**
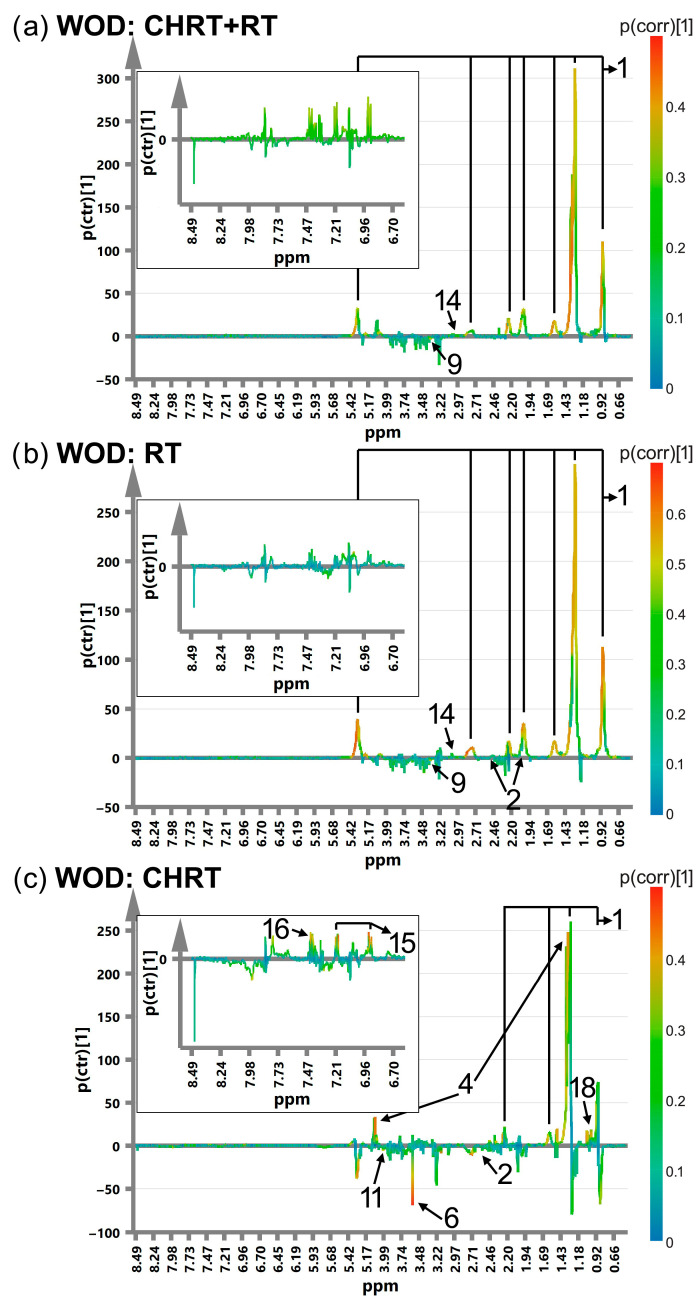
The OPLS-DA s-line plots with the indicated metabolites responsible for discrimination between the L (Low) and H (High) WOD classes for CHRT+RT (**a**), RT (**b**) and CHRT (**c**). The numbering of the metabolites corresponds to that used in Table 4. 1—lipids, 2—glutamine, 4—lactate, 6—glycine, 9—methanol, 11—serine, 14—lysine, 15—tyrosine, 16—phenylalanine, 18—valine. The corresponding cross-validated score plots are presented in Figure S2.

**Figure 2:**
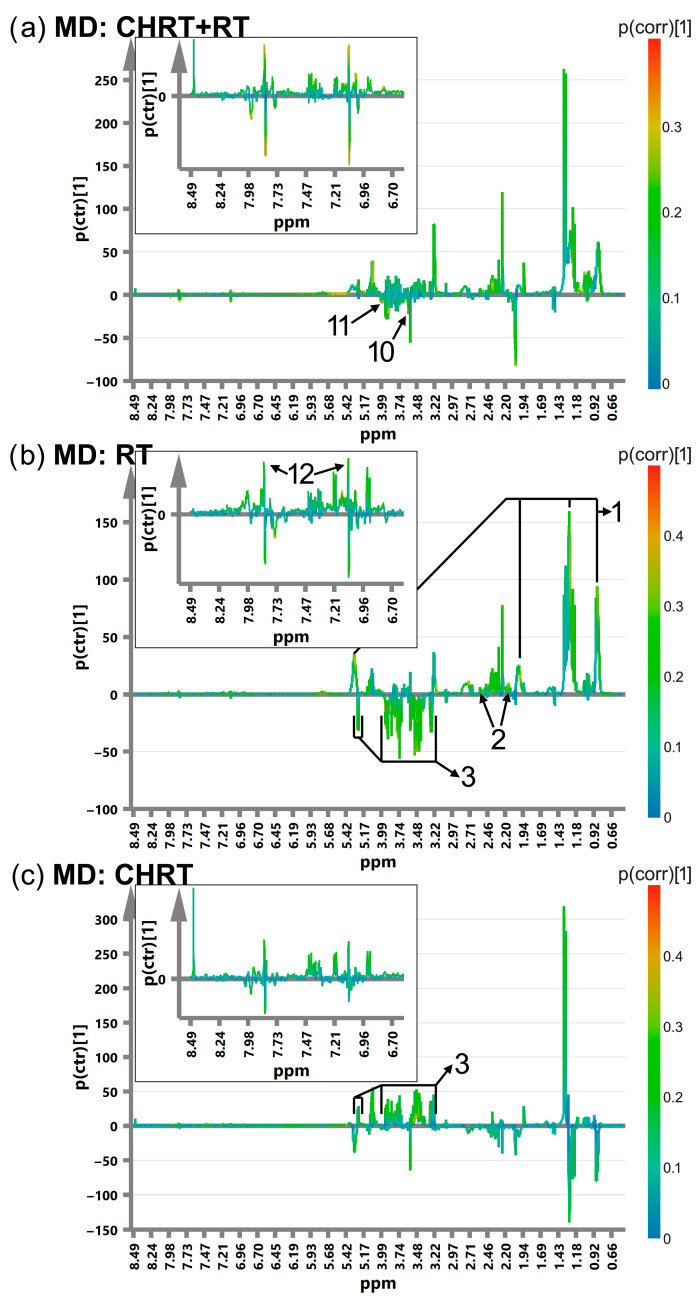
The OPLS-DA s-line plots with the indicated metabolites responsible for discrimination between the L (Low) and H (High) MD classes for CHRT+RT (**a**), RT (**b**) and CHRT (**c**). The numbering of the metabolites corresponds to that used in Table 4. 1—lipids, 2—glutamine, 3—glucose, 10—threonine, 11—serine. The corresponding cross-validated score plots are presented in Figure S3.

**Figure 3:**
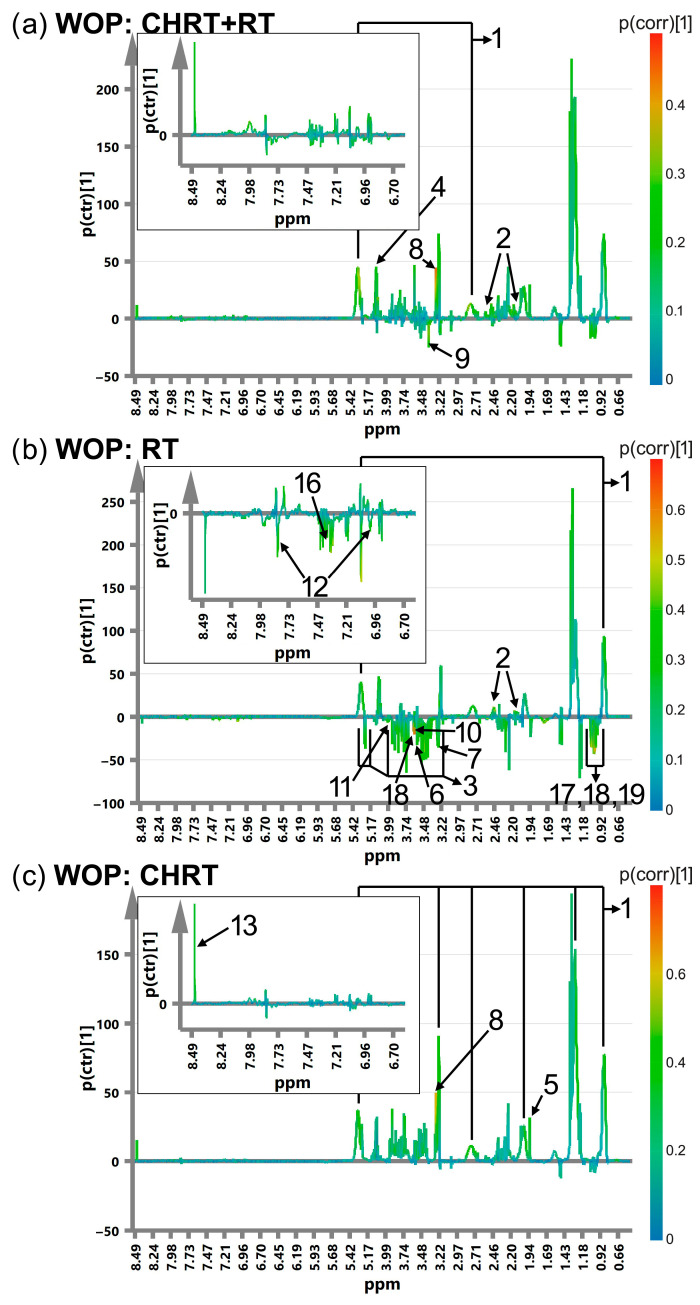
The OPLS-DA s-line plots with the indicated metabolites responsible for discrimination between the L (Low) and H (High) WOP classes for CHRT+RT (**a**), RT (**b**) and CHRT (**c**). The numbering of the metabolites corresponds to that used in Table 4. 1—lipids, 2—glutamine, 3—glucose, 4—lactate, 5—acetate, 6—glycine, 7—choline, 8—betaine, 9—methanol, 10—threonine, 11—serine, 12—histidine, 16—phenylalanine, 17—leucine, 18—valine, 19—isoleucine. The corresponding cross-validated score plots are presented in Figure S4.

**Figure 4:**
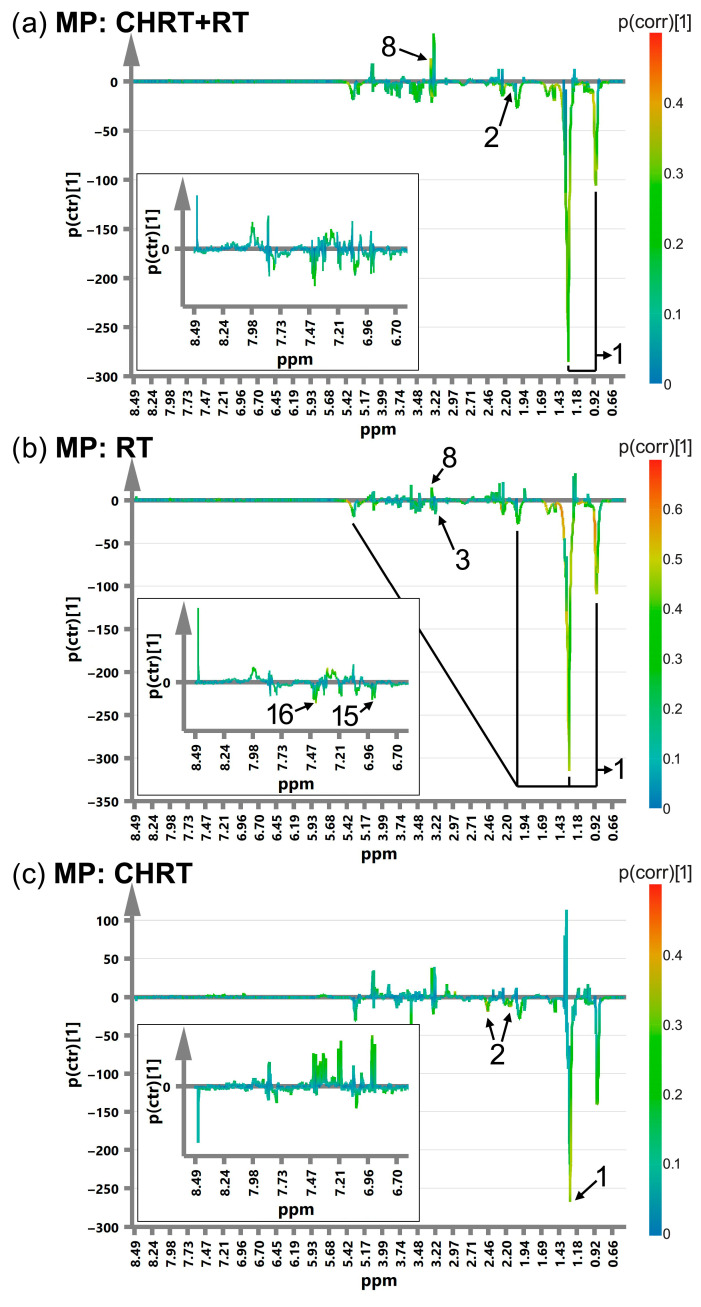
The OPLS-DA s-line plots with the indicated metabolites responsible for discrimination between the L (Low) and H (High) MP classes for CHRT+RT (**a**), RT (**b**) and CHRT (**c**). The numbering of the metabolites corresponds to that used in Table 4. 1—lipids, 2—glutamine, 3—glucose, 8—betaine, 15—tyrosine, 16—phenylalanine. In contrary to the previous analyses, a higher MP is characterized by the decreased lipid signals. The corresponding cross-validated score plots are presented in Figure S5.

Table 3 shows the quality parameters of the particular OPLS-DA models. Low predictive R2X (an amount of variation in the data that is correlated to a class separation) is usually observed in large-scale NMR data (3541 variables for a single NMR spectrum; see Section 2.6). R2Y (a total sum of variation in Y explained by the model) is mostly >0.5, reflecting a decent separation between the classes (Figure 1, Figure 2, Figure 3 and Figure 4). Two models have Q2 < 0.3, suggesting a possible low predictive ability or possible overfitting; thus, in such cases, a biological interpretation needs to be made with caution. Overall, the lowest R2Y and Q2 were observed in the CHRT+RT models (except WOP), presumably due to a slightly different metabolic response in the case of RT and CHRT (Table 3).

**Figure 5:**
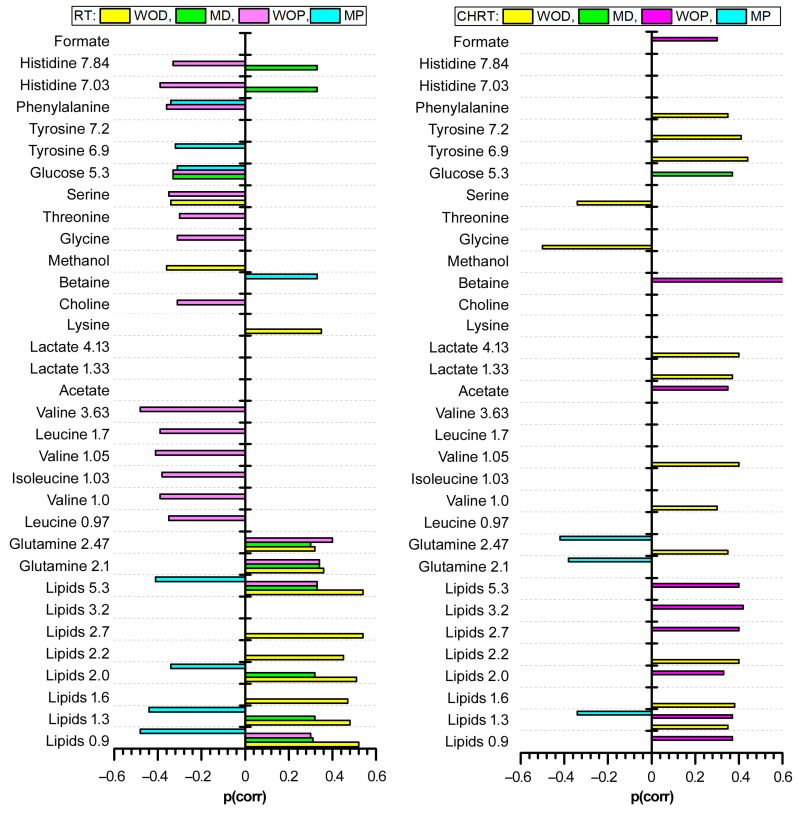
Comparison of metabolic alterations in the analyzed subgroups (WOD, MD, WOP and MD) for RT and CHRT. The length of bar corresponds to the value of the *p*(corr) parameter from OPLS-DA.

Figure 1, Figure 2, Figure 3 and Figure 4 show the OPLS-DA s-line plots, based on which the identification of the important (for discrimination) metabolites is made for WOD, MD, WOP and MP, consecutively. The corresponding cross-validated score plots discriminating the L and H classes are presented in Figures S2–S5 (in Supplementary Material). In most cases, at least a decent distinction between the classes is observed, corresponding with the R2Y values shown in Table 3. However, the number of the significant metabolites varies between the OPLS-DA models, especially for MD and MP, where for particular treatment modalities only a few or even one metabolite is relevant (Table 4). The strongest impact on the blood serum metabolic profile (expressed in the number of the altered metabolites as well as in the magnitude of *p*(corr) (the total sum of variation in Y explained by the model) is observed in WOD and WOP (Table 4). The majority of the significant metabolic alterations are related to the lipid compounds, glutamine, betaine, serine and glucose. The metabolic responses for RT and CHRT vary; therefore, the results for the whole study group will not be discussed in detail and are provided for comparative purposes only. Comparison of metabolic alterations in the analyzed subgroups (WOD, MD, WOP and MD) for RT and CHRT is presented in Figure 5.

The patients who experienced a prolonged stress (high WOD) showed significantly increased serum lipids, glutamine and decreased serine. In addition, those treated with RT had elevated lysine and a decreased methanol signal at 3.38 ppm, while in those treated with CHRT, higher levels of valine, lactate, tyrosine and phenylalanine as well as decreased levels of glycine and serine were observed. In contrast, among the patients with long-term pain (high WOP), the response of the lipid signals was much weaker, with their increase mainly in the patients treated with CHRT; this effect was accompanied by elevations of acetate, betaine and formate, while prolonged pain in the group treated with RT alone was characterized by increases in glutamine and lipid signals (only at 0.9 and 5.3 ppm) as well as by decreases in BCAAs, choline, glucose, glycine, histidine, phenylalanine, threonine and serine. The distress intensification (high MD) had almost no impact on the blood serum profile of the patients treated with CHRT; the only effect was the increase in the glucose signals, while in the case of the RT modality a higher distress resulted in increased lipids, glutamine, histidine and decreased glucose.

The elevated lipid signals and glutamine were found to be associated with the duration of distress and pain as well as with the distress magnitude (Table 4). Surprisingly, a contrary association is observed for the pain magnitude. Similarly as in WOD and MD, the impact of the pain magnitude on the blood serum metabolic profile is weaker than in the case of the pain duration (Table 4). Nevertheless, decreased lipid signals, glucose, tyrosine and phenylalanine accompanied by increased betaine characterize the high MP patients treated with RT, while those treated with CHRT show decreased glutamine and lipids at 1.3 ppm (Table 4).

Additionally, a Mann–Whitney U (MWU) test with a Bonferroni correction was used for evaluation of statistical significance of the metabolites identified as statistically important by OPLS-DA. The low-molecular-weight metabolites were quantified based on the 1D positive projections of the J-resolved spectra. The lipid signals were quantified based on the diffusion-edited spectra. The integrals were measured in the spectral regions defined individually for each metabolite using “the sum all points in region” method in AMIX (Bruker Biospin) software version 4.0.2. In Table 4, the statistically significant differences (with *p* value < 0.05) assessed by the MWU test (with a Bonferroni correction) are denoted in bold. The obtained results indicate that the influence of distress and/or pain on the blood serum metabolic profile is very subtle compared to, e.g., RT/CHRT. This is particularly visible in Table 3 in the values of R2X orthogonal (a value of 1 represents 100% of variation)—an amount of variation in the data that is uncorrelated (orthogonal) to the class separation, and therefore is removed during the orthogonal filtering in the first step of OPLS-DA. Consequently, the results obtained on the filtered data may not correspond to the results obtained on the unfiltered data. This is one of the reasons why the results obtained in multivariate projection techniques and classical statistical analyzes may not be consistent with each other.

### 3.2. Correlations between Distress and Pain

Finally, the Spearman’s rank correlation coefficients (R) were calculated to examine the relationships between WOD, MD, WOP and MP. The correlation coefficients were calculated exclusively based on the values from the distress/pain thermometer; thus, no NMR information was taken into account. The results are presented in Table 5. The features that are common for the RT and CHRT treated groups are the positive correlations between:

- The duration (WOD) and the intensity (MD) of the distress;
- The duration (WOP) and the intensity (MP) of the pain;
- The intensity of the distress (MD) and the pain (MP).

In addition, among the patients treated with RT, the duration of the distress (WOD) and the pain (WOP) are positively correlated, whereas in CHRT a positive correlation is observed between the magnitude of the pain (MP) and the duration of the distress (WOD).

**Table 5:** Results of the Spearman’s correlation between WOD, MD, WOP and MP.

R	RT				CHRT			
	WOD	MD	WOP	MP	WOD	MD	WOP	MP
WOD		**0.42**	**0.35**	0.18		**0.57**	0.23	**0.36**
MD			0.20	**0.48**			0.29	**0.59**
WOP				**0.32**				**0.45**

Spearman’s rank correlation coefficients (R). All correlations are significant with *p* < 0.05. The values with R > 0.3, indicating a moderate correlation, are in bold.

## 4. Discussion

Radiotherapy and sequential and/or concurrent chemotherapy are the standard organ preservation treatment methods for HNSCC. Despite several advancements in the curative treatment of this disease, the majority of HNSCC patients still experience substantial treatment-related adverse effects. Moreover, head and neck cancers have higher rates of emotional distress than for other cancer types and in the general population, and pain caused by the disease and its treatment is common and most debilitating. Distress and pain are associated with a poor outcome in physical, mental and social life domains [52,56]. As the disease progresses, there is an additional stressor: uncertainty faced by the patients. If the resolution of uncertainty is not possible, the ongoing brain-energy crisis leads to an allostatic load that contributes to systemic and brain pathology called “toxic stress” [57]. Such toxic distress, both as an acute and chronic condition, has a significant negative impact on the course and outcome of the oncological treatment [58]. That is why monitoring of the oncological patients, their psychological support and effective management of the distress are so important and highly desirable. The clinical approach based on the questionnaires and/or the interviews is the most common in this area due to its ease of use and little or no cost. However, this type of data is often burdened with the bias formed from the patients’ personal perspective. Therefore, independent additional markers are desirable to objectify the assessment of the patient’s psychological state. The stress response is one of the most highly conserved and fundamental biological processes in living creatures. Many different types of signals, including anxiety, depression, pain, and fear, can activate stress response pathways, and, in turn, the adrenergic signaling pathways may alter tumor metabolism. The present study is a preliminary approach to identify the ^1^H NMR detectable blood serum biomarkers of distress and pain in HNSCC patients undergoing anticancer treatment. To the best of our knowledge, such an approach has not been used before. In order to identify the key metabolites responsible for the differentiation within the groups, the OPLS-DA models were established between the WOD, WOP, MP and MD groups of patients. Because our previous research showed that the serum NMR metabolic profiles vary between the treatment modalities [45], in the present study the results are presented for the whole study group as well as for the sub-groups treated with CHRT or RT. We found that the lipid signals and those of glutamine, leucine, valine, isoleucine, acetate, lactate, lysine, choline, betaine, methanol, glycine, threonine, serine, glucose, tyrosine, phenylalanine, histidine and formate are the main NMR biomarkers of pain and/or distress in the studied groups (Table 4).

### 4.1. The Altered Metabolites Grouped According to Their Class and/or Participation in Specific Metabolic Processes

#### 4.1.1. Lipids

Lipids are a class of water-insoluble metabolites, and despite their remarkable heterogeneity most lipids are composed of common building blocks such as fatty acids (FAs) and cholesterol. They represent a diverse array of molecules essential to the cell’s structure, defense, energy and communication. Several reports have demonstrated that the consequences of a lipid metabolism deregulation in cancer not only sustain tumor growth but also promote cell migration, invasion, and angiogenesis [59]. During cancer therapy (for instance, chemotherapy and radiation therapy), a targeted inhibition of cell proliferation can likewise cause widespread and drastic changes in lipid composition [60]. As revealed by our results, the blood serum metabolic profiles of the HNSCC patients are significantly affected by the anticancer radio- or chemoradiotherapy, the lipid part of the profiles being influenced mainly by inflammation, the immune response and from fasting and ketosis [45,46,47,48].

As far as the influence of the distress and/or pain on the NMR lipids profiles is concerned, the serum lipids seem to be an important marker of the dysfunctional lipid pathways: the lipid levels increase in the patients with higher WOD CHRT and RT, MD RT, WOP CHRT and RT (although the lipid profiles vary by group) and decrease in those with higher MP (Table 4).

The relationship between dyslipidemia and distress and/or pain has been studied for decades, but the exact mechanisms of their interactions still remain unknown. In numerous studies, a significant dyslipidemia (increase in total cholesterol, triglyceride, low-density lipoprotein cholesterol and decrease in high-density lipoprotein cholesterol) is reported to occur under various stress conditions [37,38,61,62,63]. It has been postulated that the stress-induced dysregulation of the HPA axis promotes lipid production and accumulation and also enhances lipolytic hormone decomposition [64]. The disturbing effect of isolation stress is reported in an animal study [65], whose authors found altered gene expression in mice livers after 30 days of isolation. A notable feature of the gene expression profile was the involvement of the lipid metabolism pathway, including an enhancement of fatty acid synthesis and a decrease in fatty acid degradation and endoplasmic reticulum stress. The observed alterations were claimed to lead to an increase in triglycerides and a decrease in free fatty acids and ketone bodies in the circulating blood [65]. Moreover, untargeted metabolomics identified the dysregulation of sphingomyelin–ceramide metabolism during chronic neuropathic pain [66].

In our study, in an early RT treatment (within 0–20 Gy) of the HNSCC patients, the pain is considered as nociceptive; however, with increasing RT-induced mucositis the neuropathic mechanisms begin to play a more and more important role [67]. Inflammation and hyperlipidemia are known to be interconnected processes, and their underlying mechanisms are already well explained [68]; hence, the increased lipids in the high WOP patients are expected (Table 4). Surprisingly, the patients with a higher maximum pain (MP) show lowered lipid signals, in contrast to the non-cancer patients, where a positive correlation between the pain intensity and the levels of the circulating lipids is reported [69,70]. In the mentioned works, however, the pain triggers were of different etiologies and the distress factor was not taken into account. As the pain states are diverse in their respective pathophysiological mechanisms, the contribution of the specific lipid mediators to each pain state can also vary. Likewise, the cellular origin of these lipids may differ in each pain state. Among the lipids, there are membrane-derived phospholipids and oxidative metabolites of polyunsaturated fatty acids (PUFAs); some lipids may be synthesized and released as part of the inflammatory response and others upon oxidative or toxic stress [71]. Thus, the enormous chemical diversity of lipids and their dynamic systemic changes make detailed analysis and understanding of the metabolic network that the lipids are involved in very difficult [72]. Optimization and standardization of the 1H NMR measurement protocols are of paramount importance for reliable, accurate, and comparable quantifications. In order to fulfill these strong quality requirements, a high-resolution, typically 600 MHz NMR spectrometer should be used to acquire the 1H NMR spectra of blood plasma or serum samples.

#### 4.1.2. Glutamine, Glucose and Other Metabolites of Energy Metabolism

Given that carbohydrate, lipids, and proteins are the three basic energy substances, their metabolism is closely associated with energy metabolism and the citrate cycle (TCA cycle or Krebs cycle), and their shared metabolic pathways play an important and crucial role in the energy metabolic network. Glutamine, the most abundant amino acid in the human body, is involved in various aspects of biosynthesis, bioenergetics and in participating in the TCA cycle [73]. The concentration of glutamine in plasma is determined by its relative rates of release into and uptake from plasma by various tissues, such as skeletal muscle, lungs, and adipose tissue. It is also considered an essential fuel (along with glucose) for cancer cells, which consume glutamine at a rate exceeding its biosynthesis [73]. Glutamine is an important metabolic fuel that helps rapidly proliferating cells to meet the growing demand for ATP, biosynthetic precursors, and reducing agents.

We observe the increased serum glutamine levels in the high WOD (after RT), MD (after RT) and WOP (after RT) groups as well as its decrease in the high MP patients (after CHRT). It is widely reported that the circulating glutamine levels are reduced during the majority of pathological processes, including inflammation and mucositis (the major side-effects of anti-cancer treatment), because of glutamine’s utilization as an energy substrate for leukocytes as well as in the tissue repair processes and in the intracellular pathways associated with pathogen recognition [74]—hence the reduced glutamine levels in the patients with high MP. On the other hand, elevated glutamine has been observed in patients with chronic depression, suggesting disturbances in energy metabolism [75]. Muscle is the primary glutamine-producing tissue, and increased blood glutamine levels appear in response to starvation, increased proteolysis and amino acid catabolism in skeletal muscle [76]. The literature does not provide a direct explanation for the altered glutamine levels and their relationship to increased stress and pain. It can only be assumed that the higher glutamine levels in the patients with prolonged distress and pain result either from the impaired utilization of this metabolite, altered energy metabolism or the increased production of glutamine in the muscle tissue.

Glucose, the primary source of energy for living organisms, is not affected by the distress duration, but its reduced levels are observed in the RT-treated patients with prolonged and intensified pain (Table 4). The effect of the distress severity on glycemia was found in our study to depend on the treatment method: reduced levels of glucose are observed in the patients that undergo RT, elevated ones in those from the CHRT group. A decrease in glucose (although without statistical significance) has been previously reported by us in the HNSCC patients suffering from a high acute radiation sequelae (ARS) [48].

Two other metabolites involved in energy metabolism are lactate and acetate. Lactate is always an inevitable end product of glycolysis, regardless of oxygen availability, and the metabolite of energy metabolism and signaling molecules, elevated in blood during many pathological processes [77], including psychological stress, suggesting a possible increased cerebral energy demand [78]. We observe increased lactate in the high WOD group treated with CHRT as well as in the high WOP patients (without the division into the treatment modalities) (Table 4). An acetate concentration is usually increased in a starving condition due to increased beta-oxidation of adipose tissue fatty acids, and it has been observed in HNSCC patients with a high treatment-induced toxicity [48]. In the present study, the patients with prolonged pain (high WOP group) and treated with CHRT show elevated acetate (along with increased betaine, formate and the lipid signals; Table 4).

#### 4.1.3. Metabolites of One-Carbon Metabolism

One-carbon metabolism (OCM) is the process by which one-carbon groups at different oxidation states are used in a set of interconnected biochemical pathways driven by folate and homocysteine-methionine cycles, involved in DNA synthesis through purine and thymidylate generation, amino acid homeostasis, antioxidant generation, and epigenetic regulation. Remethylation of homocysteine to methionine allows several transmethylation reactions. Folate metabolism plays at least two separate roles. Moreover, it plays a role in the catabolism of choline and at least three amino acids: histidine, serine and glycine. These biochemical processes, in turn, support critical cellular functions such as cell proliferation, mitochondrial respiration and epigenetic regulation [79,80]. OCM consists of the interconnected metabolic pathways (folic acid cycle, methionine remethylation and transsulfuration), and a disruption of one of its parts affects the entire OCM.

As our study reveals, prolonged distress and/or pain seem to be reflected in disturbed OCM through the decreased levels of various metabolites, such as glycine (WOD CHRT and WOP RT), serine (WOD RT, WOD CHRT, MD CHRT+RT and WOP RT), histidine (WOP RT), threonine (WOP RT and MD CHRT+RT), methanol (WOD CHRT+RT, WOD RT and WOP CHRT+RT), choline (WOP RT), elevated betaine (WOP CHRT+RT and WOP CHRT as well as MP CHRT+RT and MP RT), formate (WOP CHRT) and histidine in MD RT. These metabolites are involved in OCM, which, in turn, has been suggested to play a role in the pathology of depression [75] and mental illness [81], among others.

Our findings indicate that the OCM metabolites are mainly lowered under prolonged distress and/or pain, except betaine and formate as well as histidine (in MD RT), which are increased (Table 4). Betaine serves as an effective protectant against neuroinflammation and oxidative stress in the pathophysiology of neurodegenerative disorders and memory impairment and also plays a major role in improving the moods of individuals with mild-to-moderate depression [82]. The betaine levels, along with those of choline, are reported to be higher in patients with chronic depression than in those with episodic major depressive disorder, although these differences were found to disappear after correction for multiple testing [75]. It is in line with the observation by Miao et al. [83], who found that in patients with post-stroke depression (PSD), the betaine and choline levels were higher than in those without PSD. However, as revealed by the results of the large population–based study by Bjelland et al. [84], choline concentrations in the blood were negatively associated with anxiety symptoms, but no significant associations were found in the case of depression. Moreover, basic science studies have demonstrated a potential anti-inflammatory and analgesic effect of choline [85]. In our study, the choline levels are decreased in the high WOP group treated with RT; thus, lower choline may be responsible for a greater sensitivity to pain in this group. However, the works on this topic are sparse, and it is difficult to draw firm conclusions other than that systemic choline concentrations may be affected.

In turn, formate, which in our study increased only in WOP CHRT, is reported as one of four metabolites that appear to follow a trend of upregulation in the biofluids of the depressed patients as measured using chromatography/nuclear magnetic resonance/mass spectrometry (the others are: glutamate, alanine and citrate) [86]. It should be remembered that pain and fatigue are the main features of chronic depression [87].

As far as glycine is concerned, its levels in plasma of patients suffering major depression are revealed to be lower when compared to the healthy controls [88]. Moreover, these authors observed no significant differences in histidine, serine, glutamine or aspartate between the major depressed and healthy subjects.

When analyzing the metabolic profiles of the studied groups it should be, however, taken into account that the OCM disturbances affect not only the metabolites involved directly or indirectly in OCM, but may also have wider negative consequences in human metabolism, inter alia interfering with lipid and fatty acid profiles [81].

#### 4.1.4. Metabolites of Protein Metabolism and Oxidative Stress

Tyrosine and phenylalanine are useful markers of protein metabolism [89] and are involved in oxidative stress [90]. Tyrosine is synthesized from phenylalanine and then becomes converted into catecholamine neurotransmitters. The relationship between mental stress and oxidative stress has been studied for decades, and while the exact mechanisms are still not fully elucidated, there is no doubt that both acute and prolonged mental stress increase oxidative stress, with HPA axis activation being involved [91,92]. In the present study, tyrosine and phenylalanine are both increased in the high WOD CHRT patients. This is in line with the study by Hu et al. [93]; in this 1H NMR-based metabolomic approach, the disturbances (increases) of phenylalanine, tyrosine, 1-methylhistidine, 3-methylhistidine and LDL CH_3_-(CH_2_)_n_- were identified in the plasma as being responsible for distinguishing the post-stroke depression (PSD) from the non-PSD subjects. These five metabolites are involved in monoamine neurotransmitter metabolism (phenylalanine and tyrosine), and in oxidative stress (1-methylhistidine, 3-methylhistidine and LDL CH_3_-(CH_2_)_n_-).

A decrease in tyrosine and phenylalanine is observed by us in the high MP RT group, while phenylalanine alone is reduced in the high WOP RT group. In the case of a complex regional pain, no statistically important changes in phenylalanine were observed [94,95]; the cited blood studies were carried out using high-performance liquid chromatography (HPLC) with fluorimetric detection. The authors reported pronounced increases in the amino acid levels, like those of glutamate, glutamine, glycine, taurine and arginine in the patients as compared to the controls.

The link between pain perception and the metabolism of phenylalanine and tyrosine requires a separate study, because there are practically no literature reports on this issue.

Lysine, an exogenous metabolite, is increased in the high WOD group. The main role of this metabolite is participation in protein synthesis and glutamate formation; it is also a precursor for the biosynthesis of carnitine [96]. Therefore, elevated lysine may result from attenuation of these metabolic processes under prolonged distress. On the other hand, lowered blood lysine was observed in patients with major depressive disorder [97] and in healthy volunteers experiencing mental fatigue [98].

#### 4.1.5. Branched-Chain Amino Acids (BCAAs)

Although BCAAs (leucine, valine and isoleucine) are involved, inter alia, in energy and protein metabolism, they are often discussed as a separate class of amino acids because, unlike most amino acids, they are catabolized in skeletal muscle and not in the liver. The branched-chain amino acids have been found to be involved in stress, energy, and muscle metabolism and also have neurophysiological therapeutic effects related to the competitive relationship with aromatic amino acids responsible for the release of several neurotransmitters, notably serotonin (from tryptophan), and catecholamines (from phenylalanine and tyrosine) [99].

In our study, BCAAs are markedly reduced in the RT-treated patients suffering from a prolonged pain (high WOP RT), whereas in WOD CHRT only valine increases (Table 4). The BCAAs reduction is in line with our previous study on anticancer treatment toxicity in HNSCC [48]. During a short-term fasting or starvation, muscle protein deposit is catabolized for energy, resulting in elevated BCAAs in the blood. However, after a longer period of time, the BCAAs levels decrease due to depletion of muscle protein [100].

In depressed subjects, the discrepancy in the directions of change in the amino acid levels for major depressive disorder patients exists across multiple studies [101]. However, the BCAAs—valine, leucine and isoleucine—are often claimed to be significantly decreased in patients with major depression [102]. The upregulation of BCAAs is, in turn, claimed to contribute to reduction in the uptake of tryptophan and the synthesis of serotonin and may play a role in delaying central fatigue [103].

### 4.2. Correlations between the Analyzed Groups

Table 5 shows the correlation of distress and/or pain (duration and intensity) among patients treated with RT and CHRT. We observe a clear positive correlation between the severity and duration of both pain and distress; nevertheless, the metabolic characteristics between WOD and MD and WOP and MP, although similar in the RT modality, do not completely overlap or differ significantly in CHRT (Table 4). It is worth noting that the intensity of distress and/or pain exerts a negligibly small effect on the metabolic profile of patients treated with CHRT (Table 4). On the other hand, chemotherapy itself has a significant impact on the blood serum metabolic profile [45,46] and, thus, may hinder finding more subtle alterations.

### 4.3. Limitations of the Study

We are aware that the above study is subject to a number of factors that may affect the results obtained. However, this is a pilot study, analyzing the possibilities of understanding the impact of stress and pain during anticancer therapy on the metabolic profile of blood serum. The obtained results may allow for further research focused on specific aspects or narrower subgroups of patients.

Although the total group consists of almost 70 patients, it is quite heterogeneous in terms of gender, tumor location and stage. Head and neck cancers are more frequent in men, and therefore women are insufficiently represented in the present study. It can therefore be assumed that the results obtained are characteristic mainly for the male population. The possible variation in the serum metabolites between men and women was not taken into account in the present study. Our previous studies [45,46,47,48] show that in the case of HNSCC tumors of homogeneous etiology, the location of the tumor has a negligible impact (for the methods used here) on the metabolic profile. The difference in the degree of disease advancement (and ultimately in the volume irradiated due to lymph node involvement) is already reflected in the division into the RT and CHRT subgroups [45].

The analyzed metabolic profile is affected by several pathological stimuli, including the patient’s systemic response to the treatment and the local response of the tumor. Thus, visualization of more subtle metabolic changes related to a patient’s psychological status is difficult. Finally, distress and/or pain are positively correlated (Table 5), making it impossible to clearly distinguish whether the overlapping metabolic alterations are due to distress, pain or both.

The patients presented no comorbidities that could significantly alter the metabolic profile and/or distress and pain during the anticancer treatment.

## 5. Conclusions

This preliminary study sheds a light on a metabolic response correlated with the du-ration and magnitude of distress and/or pain in HNSCC patients undergoing anti-cancer therapy. Although the study is burdened by several important limitations, the preliminary results indicate that distress and/or pain:

- Primarily affect plasma lipids, and this effect, seen as an increase in the integral in-tensities of the lipid signals, is particularly intense during prolonged stress (OPLS-DA *p*(corr) values from 0.35 to 0.54);
- Disturb energy metabolism by strong alterations in the glutamine levels;
- Impact one-carbon metabolism (the prolonged distress and pain reduce the levels of glycine, serine and methanol, the intensified distress and prolonged pain alter the levels of threonine and histidine, while the intensified pain increases the levels of betaine).

A strong (OPLS-DA *p*(corr) values from −0.35 to −0.48) negative impact of the pro-longed pain on the branched-chain amino acids as well as sparse disturbances in the metabolites of protein metabolism and oxidative stress (lysine, tyrosine and phenylalanine) are also observed.

A particularly interesting finding is the reversed metabolic response to severe pain (the MP subgroup) compared to the other subgroups studied (duration (WOD) and intensity (MD) of distress and duration of pain (WOP)). The above is particularly visible in the cases of lipids, glutamine, tyrosine and phenylalanine. Intensified pain leads to a decrease in the levels of these metabolites, while in the other subgroups their signals are increased.

The results obtained show that the severity of inflammation is not directly correlated with the duration and magnitude of distress and/or pain. On the other hand, the duration and magnitude of pain share similar metabolic alterations with our previous study of treatment-induced toxicity; such a finding is reasonable, as the sensation of pain is directly related to the severity of acute radiation sequelae. Further studies in a larger and more homogeneous group of patients are necessary to resolve the influence of aggravating factors, which is the main limitation of this study.

## Figures and Tables

**Table 1:** Characteristics of the whole study group.

Treatment Modality	RT	CHRT	Between Group Difference (*p* Value from MWU or χ2 Test)
Age			0.055
Range	46–73	46–74	
Median age	61	58	
Sex			0.54
Males	26	26	
Females	10	7	
Tumor localization			0.001
Hypopharynx	3	7	
Larynx	26	14	
Nasopharynx	1	2	
Oropharynx	6	10	
Tumor staging			
T (primary tumor stage)			0.045
1	3	2	
2	22	10	
3	7	11	
4	4	10	
N (nodal stage)			0.000
0	30	10	
1	2	5	
2	4	17	
3	0	1	
TNM (tumor, nodes, metastases)			0.000
I	2	0	
II	21	2	
III	6	8	
IVa	7	22	
IVb	0	1	

Legend: MWU—Mann–Whitney U test (continuous variables); χ2—Chi-squared test (categorical variables).

**Table 2:** Distress and pain throughout the whole study group, divided into the RT and CHRT treatment modalities.

**WOD**				**WOP**			
**No. of Weeks When Distress > 0**	**No. of Patients**			**No of Weeks When Pain > 0**	**No. of Patients**		
	**Wholestudy Group**	**RT**	**CHRT**		**Wholestudy Group**	**RT**	**CHRT**
0	0	0	0	0	1	1	0
1	6	1	5	1	4	2	2
2	9	5	4	2	2	1	1
3	11	6	5	3	1	1	0
4	3	3	0	4	6	5	1
5	9	7	2	5	9	7	2
6	6	4	2	6	18	8	10
7	18	10	8	7	23	11	12
8	7	-	7	8	5	-	5
Median of weeks when distress > 0				Median of weeks when pain > 0			
	5	5	6		6	6	7
**MD**				**MP**			
**Max Distress Value**	**No. of Patients**			**Max Pain Value**	**No. of Patients**		
	**Whole Study Group**	**RT**	**CHRT**		**Whole Study Group**	**RT**	**CHRT**
0	0	0	0	0	1	1	0
1	18	6	12	1	3	2	1
2	14	10	4	2	7	2	5
3	9	5	4	3	17	8	9
4	7	2	5	4	13	9	4
5	10	8	2	5	13	6	7
6	3	2	1	6	5	4	1
7	4	2	2	7	6	4	2
8	2	0	2	8	2	0	2
9	0	0	0	9	1	0	1
10	2	1	1	10	1	0	1
Median of max distress value				Median of max pain value			
	3	3	3		4	4	4

Legend: WOD (weeks of distress)—the number of weeks during RT/CHRT when the distress was >0; MD (maximum of distress)—a maximum value of the distress during RT/CHRT; WOP (weeks of pain)—the number of weeks during RT/CHRT when the pain was >0; MP (maximum of pain)—a maximum value of the pain during RT/CHRT.

**Table 3:** OPLS-DA models quality parameters.

OPLS-DA Model Quality												
	WOD			MD			WOP			MP		
	CHRT+RT	RT	CHRT	CHRT+RT	RT	CHRT	CHRT+RT	RT	CHRT	CHRT+RT	RT	CHRT
R2X predictive	0.08	0.13	0.04	0.01	0.03	0.01	0.02	0.03	0.05	0.05	0.09	0.02
R2Y	0.54	0.65	0.62	0.37	0.51	0.63	0.50	0.30	0.66	0.43	0.56	0.47
Q2	0.41	0.49	0.52	0.23	0.31	0.43	0.32	0.17	0.53	0.30	0.39	0.32
NOOC	6	5	4	4	5	4	7	3	6	5	5	4
R2X orthogonal	0.70	0.64	0.67	0.70	0.76	0.64	0.78	0.68	0.72	0.68	0.70	0.70
cv-ANOVA *p* value	0.000	0.000	0.000	0.000	0.000	0.000	0.000	0.000	0.000	0.000	0.000	0.000

Legend: R2X predictive—an amount of variation in the data that is correlated to class separation; R2Y—a fraction of the class membership (Y) variation modeled using the data matrix (X); this parameter qualifies the separation between two classes; Q2—a predictive ability of the OPLS-DA model; NOOC—number of orthogonal components; R2X orthogonal—an amount of variation in the data that is uncorrelated (orthogonal) to the class separation; cv-ANOVA—cross-validated ANOVA.

**Table 4:** The OPLS-DA-identified metabolites important for class-discrimination.

OPLS-DA Results														
#	Metabolite	ppm	*p*(corr)											
			WOD			MD			WOP			MP		
			CHRT+RT	RT	CHRT	CHRT+RT	RT	CHRT	CHRT+RT	RT	CHRT	CHRT+RT	RT	CHRT
	Lipids													
1	Lipids	0.9	0.42	**0.52**			0.31			0.3	0.37	**−0.34**	−0.48	
	Lipids	1.3	**0.43**	**0.48**	0.35		0.32				0.37	**−0.31**	**−0.44**	−0.34
	Lipids	1.6	0.4	0.47	0.38									
	Lipids	2.0	0.4	0.51			0.32				0.33		−0.34	
	Lipids	2.2	0.4	0.45	0.4									
	Lipids	2.7	0.37	0.54					0.32		0.4			
	Lipids	3.2									0.42			
	Lipids	5.3	0.37	**0.54**			0.33		**0.33**	0.33	0.4		−0.41	
	Glutamine, glucose and other metabolites of energy metabolism													
2	Glutamine	2.1		0.36			0.34		0.32	0.34		−0.31		−0.38
	Glutamine	2.47		0.32	0.35		0.32		0.31	0.4				−0.42
3	Glucose	*					−0.33	0.37		−0.33			−0.31	
4	Lactate	1.33			**0.37**									
	Lactate	4.13			**0.4**				0.3					
5	Acetate	1.93									0.35			
	Metabolites of one-carbon metabolism													
6	Glycine	3.57			**−0.5**					−0.31				
7	Choline									−0.31				
8	Betaine	3.27							**0.46**		**0.63**	**0.34**	0.33	
9	Methanol	3.38	−0.31	−0.4					−0.31					
10	Threonine	3.6				−0.35				−0.3				
11	Serine	3.99		−0.3	−0.34	−0.34				−0.35				
12	Histidine	7.03					0.33			−0.39				
	Histidine	7.84					0.33			−0.33				
13	Formate	8.5									**0.3**			
	Metabolites of protein metabolism and oxidative stress													
14	Lysine	3.0	0.31	0.35										
15	Tyrosine	6.9			0.44								−0.32	
	Tyrosine	7.2			0.41									
16	Phenylalanine	7.4			0.35					−0.36			−0.34	
	Branched-chain amino acids (BCAAs)													
17	Leucine	0.97								−0.35				
	Leucine	1.7								−0.39				
18	Valine	1.0			0.3					−0.39				
	Valine	1.05			0.4					−0.41				
	Valine	3.63								−0.48				
19	Isoleucine	1.03								−0.38				

A positive/negative *p*(corr) value means a positive/negative correlation with the High WOD, WOP, MD and MP classes. High is referred as ≥ the median value. The patients with the values ≥ than the median in a given variable were assigned to the High class. *—more than 40 peaks constitute the glucose signals observed in blood serum. In the present analysis, only a few glucose signals showed *p*(corr) > 0.3. Bold font denotes the statistically significant (*p* < 0.05) differences assessed with a Mann–Whitney U test with a Bonferroni-corrected *p* value. In this work, multiple samples from the same patient are analyzed. Therefore, in MWU tests, the medians of relative concentrations of individual metabolites calculated from all measurement points of a given patient are used; 69 independent cases were compared. The differences in phenylalanine, lysine and histidine were not taken into consideration due to quantification error and/or overlapping with other signals of higher intensity.

## Data Availability

The datasets generated and/or analyzed during the current study are available from the corresponding author on a reasonable request. The distress and pain data show personal state of each patient and must be anonymized before being shared.

## Supplementary Materials

The following supporting information can be downloaded at: https://www.mdpi.com/article/10.3390/metabo14010060/s1, Figure S1: The 400 MHz 1H-NMR CPMG spectrum of blood serum from HNSCC patient treated with chemoradiotherapy. Main metabolites are indicated; Figure S2: Multivariate discrimination between the L (Low) and H (High) WOD classes for CHRT+RT (a), RT (b) and CHRT (c). The OPLS-DA cross-validated scores plots show distinct separation between the classes; Figure S3: Multivariate discrimination between the L (Low) and H (High) MD classes for CHRT+RT (a), RT (b) and CHRT (c). The OPLS-DA cross-validated scores plots show moderate to distinct separation between the classes; Figure S4. Multivariate discrimination between the L (Low) and H (High) WOP classes for CHRT+RT (a), RT (b) and CHRT (c). The OPLS-DA cross-validated scores plots show moderate to distinct separation between the classes; Figure S5: Multivariate discrimination between the L (Low) and H (High) MP classes for CHRT+RT (a), RT (b) and CHRT (c). The OPLS-DA cross-validated scores plots show only moderate separation between the classes; Table S1: NMR pulse sequence parameters.
