# Supplementary material for: Metabolite Biomarkers of Prolonged and Intensified Pain and Distress in Head and Neck Cancer Patients Undergoing Radio- or Chemoradiotherapy by Means of NMR-Based Metabolomics—A Preliminary Study
Source: Metabolites. 2024 Jan 17;14(1):60. doi: 10.3390/metabo14010060 (PMC10819132; doi:10.3390/metabo14010060)
Supplement: Supplementary file 1 [file metabolites-14-00060-s001.zip › metabolites-2756154-supplementary.pdf]

Supplementary material for:

Metabolite biomarkers of prolonged and intensified pain and distress in head and neck cancer patients undergoing radio- or chemoradiotherapy by means of NMR based metabolomics – a preliminary study

Łukasz Boguszewicz [1,*], Alicja Heyda [2], Mateusz Ciszek [1], Agata Bieleń [2], Agnieszka Skorupa [1], Jolanta Mrochem-Kwarciak [3], Krzysztof Składowski [2] and Maria Sokół1 [2]

1 Department of Medical Physics, Maria Sklodowska-Curie National Research Institute of Oncology, Gliwice Branch, 44-102 Gliwice, Poland; mateusz.ciszek@gliwice.nio.gov.pl (M.C.); agnieszka.skorupa@gliwice.nio.gov.pl (A.S.)

2 1st Radiation and Clinical Oncology Department, Maria Sklodowska-Curie National Research Institute of Oncology, Gliwice Branch, 44-102 Gliwice, Poland; alicja.heyda@gliwice.nio.gov.pl (A.H.); agata.bielen@gliwice.nio.gov.pl (A.B.); krzysztof.skladowski@gliwice.nio.gov.pl (K.S.); maria.sokol@gliwice.nio.gov.pl (M.S.)

3 Analytics and Clinical Biochemistry Department, Maria Sklodowska-Curie National Research Institute of Oncology, Gliwice Branch, 44-102 Gliwice, Poland; jolanta.mrochem-kwarciak@gliwice.nio.gov.pl

* Correspondence: lukasz.boguszewicz@gliwice.nio.gov.pl

The characteristics of the acquired spectra as well as the pulse sequence parameters

NOESY (Nuclear Overhauser Effect Spectroscopy) – an overview of all types of molecules.

CPMG (Carr-Purcell-Meiboom-Gill) – an information on only low molecular weight metabolites.

DIFF (diffusion edited) - mainly macromolecular signals.

Two dimensional (2D) JRES (J-resolved) – a visualization of scalar couplings and improved metabolite identification.

Table S1. NMR pulse sequence parameters.

Pulse program	NOESYGPPR1D	CPMGPR1D	LEDBPGPPR2S1D	JRESGPPRQF
**TD**	65536	65536	65536	8192
**SW** [ppm]	30	20	30	16.62
**AQ** [sec]	2.73	4.09	2.73	0.62
**D1** [sec]	4	4	4	2
**D8** [sec]	0.01	-	-	-
**D16** [sec]	-	-	0.0002	0.0002
**D20** [sec]	-	0.0003	0.12	-
**D21** [sec]	-	-	0.005	-
**DS**	4	4	4	16
**L4**	-	126	-	-
**NS**	32	64	64	1
**DELTA1** [sec]	-	-	0.11572488	-
**DELTA2** [sec]	-	-	0.004172	-

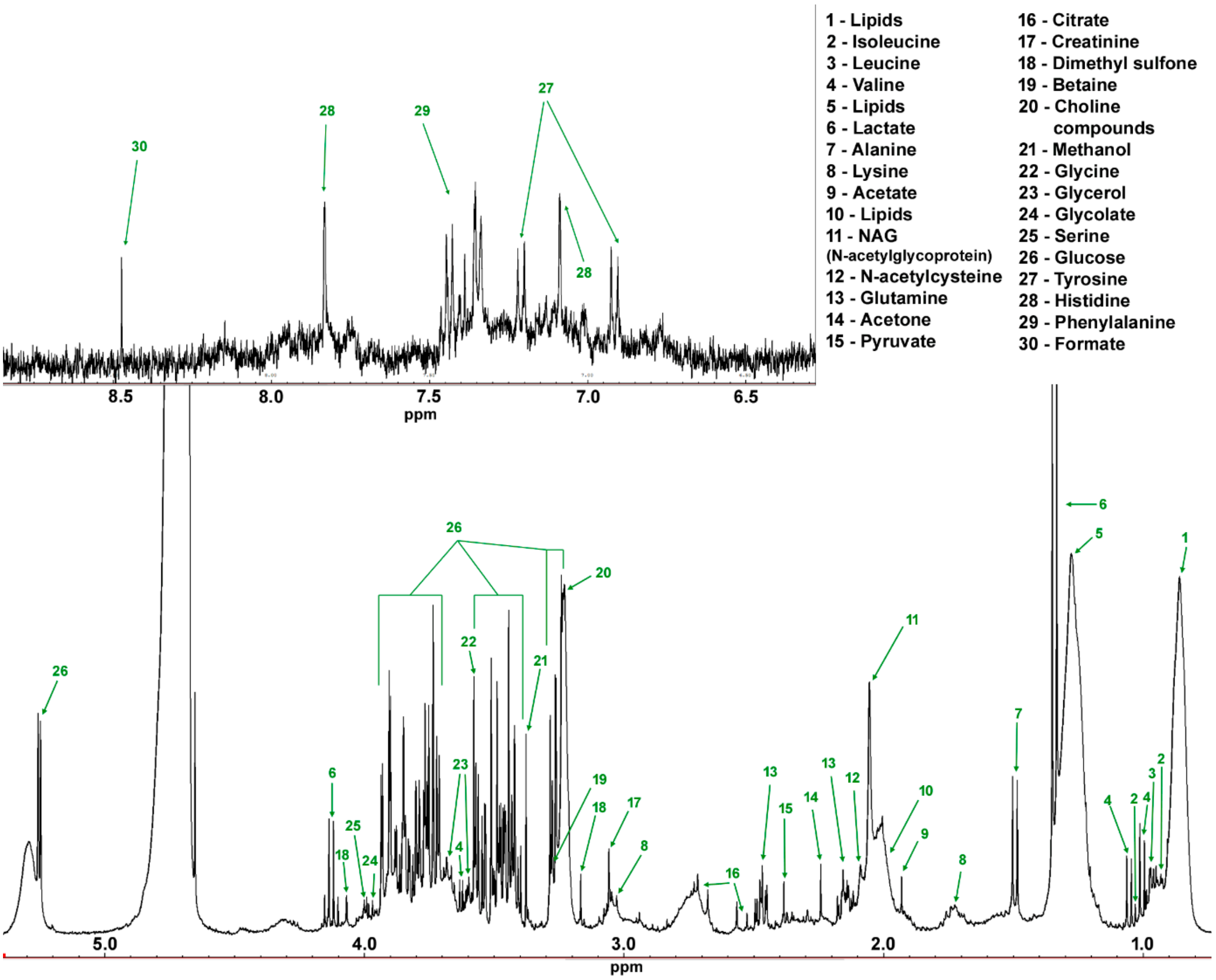

Figure S1. The 400 MHz 1H-NMR CPMG spectrum of blood serum from HNSCC patient treated with chemoradiotherapy. Main metabolites are indicated.

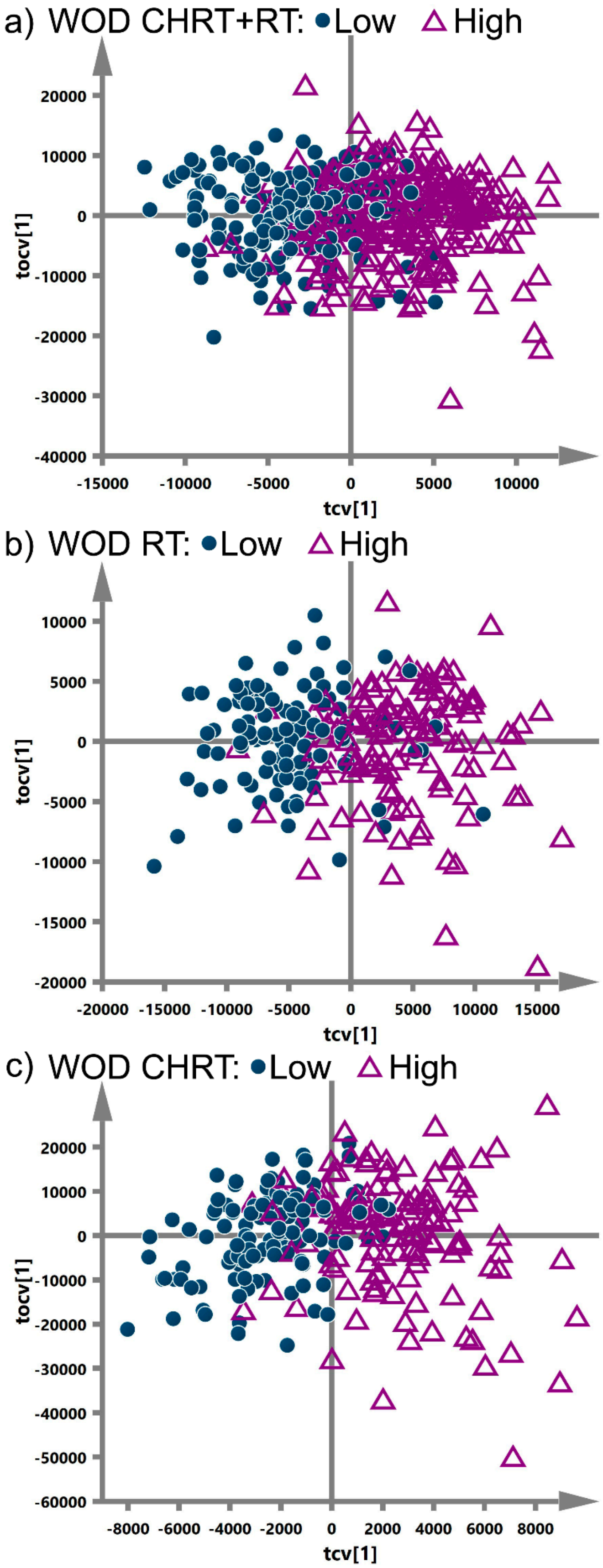

Figure S2. Multivariate discrimination between the L (Low) and H (High) WOD classes for CHRT+RT (a), RT (b) and CHRT (c). The OPLS-DA cross-validated scores plots show distinct separation between the classes.

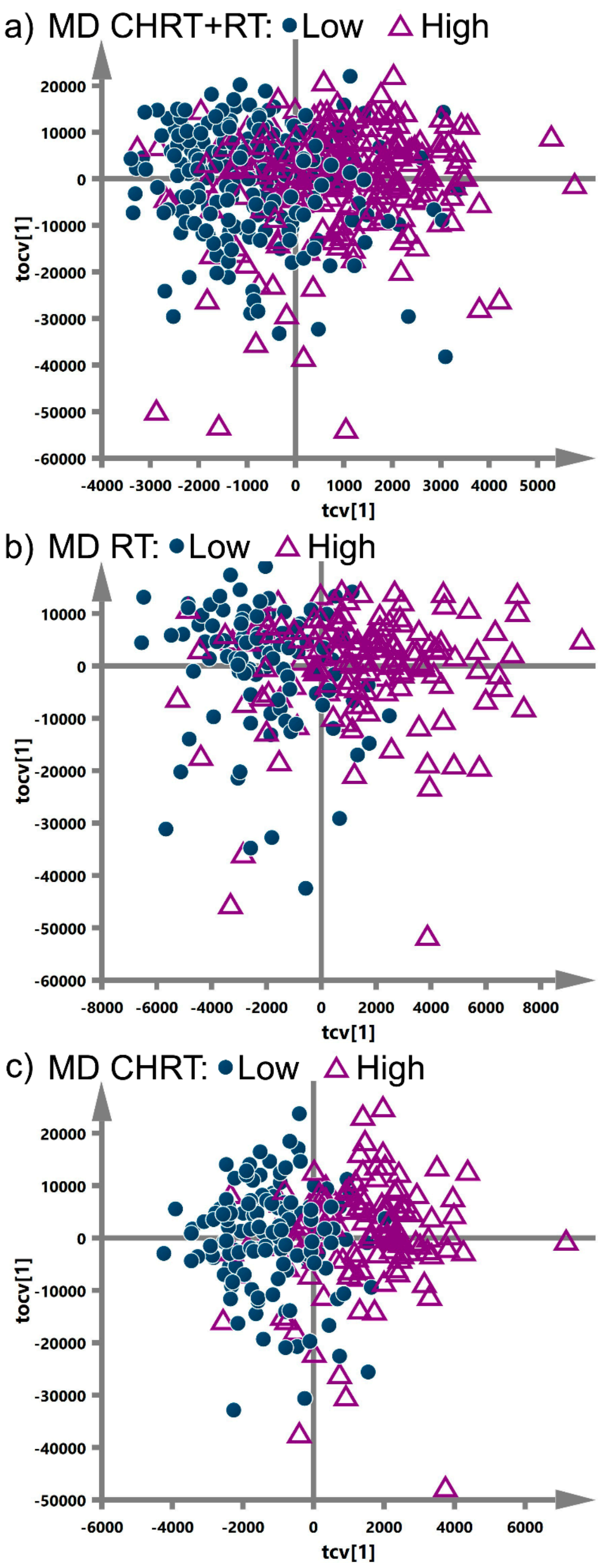

Figure S3. Multivariate discrimination between the L (Low) and H (High) MD classes for CHRT+RT (a), RT (b) and CHRT (c). The OPLS-DA cross-validated scores plots show moderate to distinct separation between the classes.

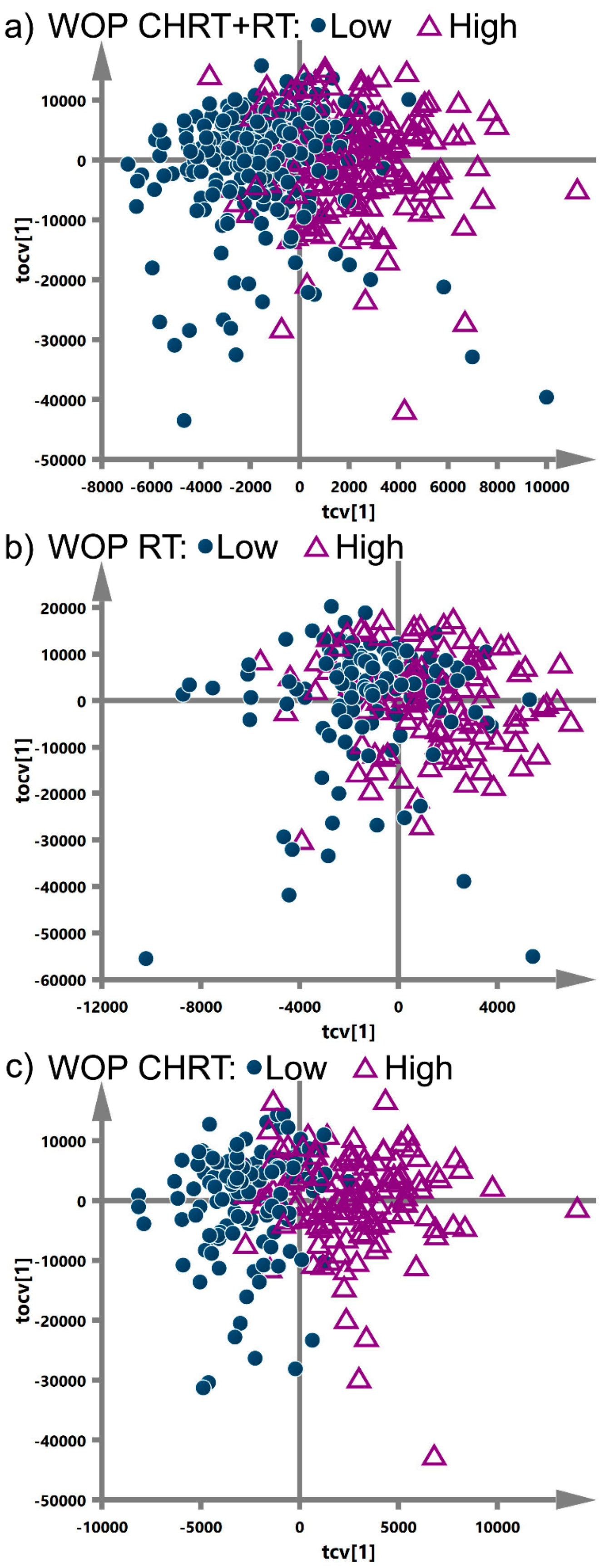

Figure S4. Multivariate discrimination between the L (Low) and H (High) WOP classes for CHRT+RT (a), RT (b) and CHRT (c). The OPLS-DA cross-validated scores plots show moderate to distinct separation between the classes.

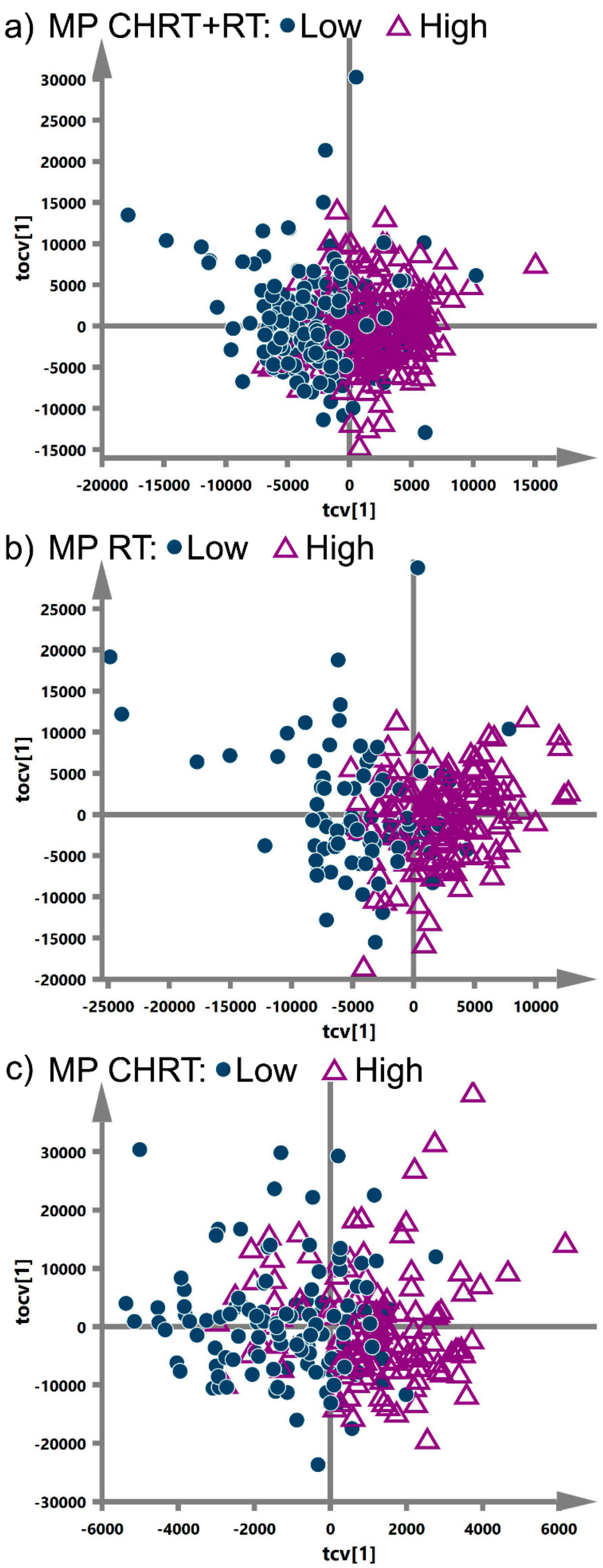

Figure S5. Multivariate discrimination between the L (Low) and H (High) MP classes for CHRT+RT (a), RT (b) and CHRT (c). The OPLS-DA cross-validated scores plots show only moderate separation between the classes.
